# Unusual skull base metastasis from neuroendocrine tumor: a case report

**DOI:** 10.1186/s13256-019-2214-5

**Published:** 2019-08-30

**Authors:** Kok Haw Jonathan Lim, Juan W. Valle, Angela Lamarca

**Affiliations:** 1 Department of Medical Oncology, The Christie NHS Foundation Trust, 550 Wilmslow Road, Manchester, M20 4BX UK; 2 Department of Medicine, Imperial College London, London, UK; 3 Institute of Cancer Sciences, The University of Manchester, Manchester, UK

**Keywords:** Endocrine tumor, Neuro-endocrinology, Neuro-oncology, Cranial nerves, Neuro-ophthalmology

## Abstract

**Background:**

With increasing treatment options available, neuroendocrine tumor has become a chronic disease and may present later on with atypical manifestation of disease spread once resistant to treatment.

**Case presentation:**

A 74-year-old white British woman undergoing treatment for metastatic well-differentiated neuroendocrine tumor for the past 9 years presented with a brief history of mild frontal headache, and progressive left ptosis and ocular palsy. She had no visual loss, and had neither speech nor motor deficit. At the outset, it was crucial to exclude acute or missed stroke. An urgent magnetic resonance imaging of her head revealed an unusual skull base metastasis extending into the cavernous sinus, with no peritumoral edema. Following discussion at a specialist neuro-oncology meeting and a neuroendocrine tumor multidisciplinary team meeting, she proceeded to have conventional fractionated radiotherapy followed by subsequent palliative chemotherapy.

**Conclusions:**

Intracranial metastasis is rare in patients with neuroendocrine tumor, particularly in those with well-differentiated histology; skull base metastasis is even more uncommon. Management of intracranial metastasis from a rare tumor should always be discussed in a specialist multidisciplinary meeting. Surgery or radiotherapy, including stereotactic radiosurgery, should be considered in skull base metastases. Hormonal abnormalities may occur following radiotherapy to skull base metastases and should be monitored closely in the first few months post treatment.

## Background

Neuroendocrine tumors (NETs) are rare neoplasms which originate from endocrine cells, predominantly found in the gastrointestinal tract and lung. Although its prevalence is estimated to be around 35 per 100,000 [1], its incidence is rising, in part due to improved histological understanding and increasing recognition of this disease entity. There are many subtypes of NETs and they are managed according to their anatomical site of origin, histological grading (grades 1–3 based on proliferation index Ki-67 and mitotic rate), stage, and clinical symptoms [2].

In England, the incidence of small intestinal NETs is 0.32 per 100,000 per year [2]. Metastases to lymph nodes and liver are common, particularly in grade 2 NETs or grade 3 neuroendocrine carcinomas (NECs), but approximately only 12% spread to the bones [3, 4]. Metastasis to the brain is very rare, with only a few small case series reporting a prevalence of up to 5%, many of which are from bronchial primary [5, 6]. Approximately half of NETs arising from the gastroenteropancreatic axis (GEP-NETs) occur in the intestine, and, in 30% of these, patients present with carcinoid syndrome (flushing, diarrhea and dyspnea) due to excess serotonin release from liver metastases [2].

There have been major advances in systemic therapies for the management of GEP-NETs in the past decade. As the majority of patients present with metastatic disease, the aims of palliative treatment are to control symptoms and tumor growth in order to maintain optimal quality of life. In fact, patients with small intestinal NETs can expect to live more than 10 years [7, 8]. In selected scenarios, it is not unreasonable to enter an initial period of “watch and wait.” For the majority of patients, first-line systemic treatment is based on somatostatin analogs (SSAs), including octreotide or lanreotide, which work as anti-proliferative agents and have been shown to significantly prolong progression-free survival (PFS) [9, 10]. More recently, the mTOR inhibitor everolimus and tyrosine kinase inhibitor sunitinib have also been confirmed to improve PFS, and are now recommended for management of advanced GEP-NETs [11]. In addition, the successful use of various combination chemotherapy regimens has been reported, particularly in cases with high disease burden, as has an increasing range of liver-directed therapies and other nuclear medicine “theranostics” [11]. The question of which would be the most efficacious sequence or combination of treatments remains unresolved.

We report the case of an elderly woman with metastatic small intestinal NET who presented with a new left ptosis. This case highlights the need for clear history and vigilant clinical examination during routine out-patient review, especially in patients who have been managed and followed-up for a long time. It also represents an interesting example of atypical disease manifestation in NET, raising awareness of the natural history of this disease when managed as a chronic disease.

## Case presentation

A 74-year-old white British woman presented to her routine out-patient appointment with a few weeks’ history of frontal headache and progressive left ptosis. She had a long history of metastatic well-differentiated grade 2 (Ki-67, 3%) non-functional small intestinal NET with known liver metastases; she was initially diagnosed 9 years ago when she underwent palliative resection for an obstructive primary tumor in the distal ileum (stage IV, T3 N1 M1). Following surgery, she started palliative treatment with systemic and locoregional therapies including (in chronological order): octreotide long-acting release (LAR) 10 mg intramuscularly every 4 weeks (PFS 3 months), transarterial chemoembolization (TACE) (PFS 5 months), everolimus 10 mg orally once daily (PFS 4 months), selective internal radiation therapy (SIRT) (PFS 11 months), lanreotide 120 mg subcutaneously every 4 weeks (PFS 5 months), and peptide receptor radionuclide therapy (PRRT) with lutetium with maintenance lanreotide (PFS 45 months). A year ago, she underwent a cytoreductive transabdominal hysterectomy and bilateral salpingo-oophorectomy for metastatic NET.

At the time of this clinic review, she was a month into her seventh-line of treatment with capecitabine 600 mg/m^2^ twice daily orally (on days 1–14) and temozolomide 150 mg/m^2^ orally divided into two doses daily (on days 10–14) of a 28-day cycle (CAPTEM), and had attended for a scheduled toxicity check. She complained mainly of drooping and swelling of her left eyelid, but denied any visual loss and did not have any other sensory or motor deficit. She had not had any recent falls or trauma.

Prior to her diagnosis of metastatic NET, she had been physically fit and well, with a past medical history of diet-controlled hypertension and previous partial thyroidectomy for benign pathology. She was not on any other regular medications and had no known drug allergies. There was no relevant family history to report. She was an ex-tobacco smoker, and drank alcohol moderately. She was a retired civil servant, lived with her husband and had two grown-up children.

On examination, she had a complete left ptosis with left ocular palsy. There was no loss of visual field, and her forehead was spared. Other cranial nerves, speech, and upper and lower limb examinations were unremarkable. She was of slim build (weight 53.4 kg), Eastern Cooperative Oncology Group performance status (ECOG PS) 1, and her vital signs (blood pressure 134/75 mmHg, heart rate 80 beats per minute, respiratory rate 18/minute, oxygen saturation 95% on room air, and temperature 36 °C), cardiorespiratory examination, and abdominal examination were normal. She had not been significantly myelosuppressed on treatment and biochemistry tests, including renal and liver function tests, were satisfactory.

On clinical examination, she presented with isolated left oculomotor nerve palsy (third cranial nerve palsy). In view of her age and history of hypertension, it was crucial to rule out a cerebrovascular event such as a posterior communicating artery stroke. As her presentation was not acute, the window of opportunity for thrombolysis had closed and, either way, her National Institutes of Health Stroke Scale (NIHSS) score was too low (< 4) for such an approach. Other considerations included demyelinating disease (multiple sclerosis) or myasthenia gravis. With a long history of metastatic disease, despite the rarity of intracranial metastasis in NET, her symptoms could be mechanically explained by the presence of a space-occupying lesion.

**Fig. 1:**
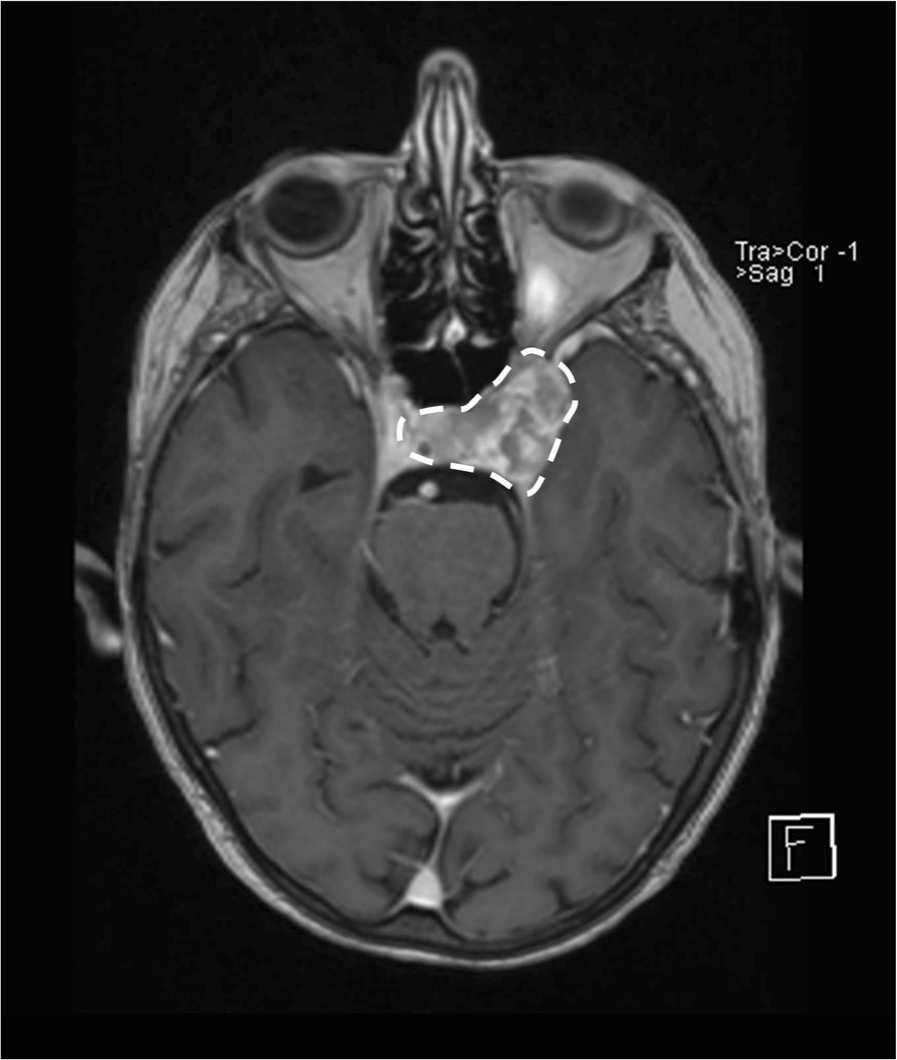
Magnetic resonance imaging of the head with contrast demonstrating an infiltrative enhancing mass in patient’s left skull base extending into cavernous sinus measuring 2.6 × 1.8 cm (encompassed within *dotted white line*). Medially, this is inseparable from the pituitary gland

An urgent magnetic resonance imaging (MRI) of her head with contrast was performed on the same day, and this unfortunately revealed a new ipsilateral skull base metastasis extending into the cavernous sinus, measuring 2.6 × 1.8 cm (Fig. 1). There was no surrounding edema, suggesting that it was not acute and may have grown over a longer period of time than the onset of her symptoms. Therefore steroids were not indicated in this instance.

**Fig. 2:**
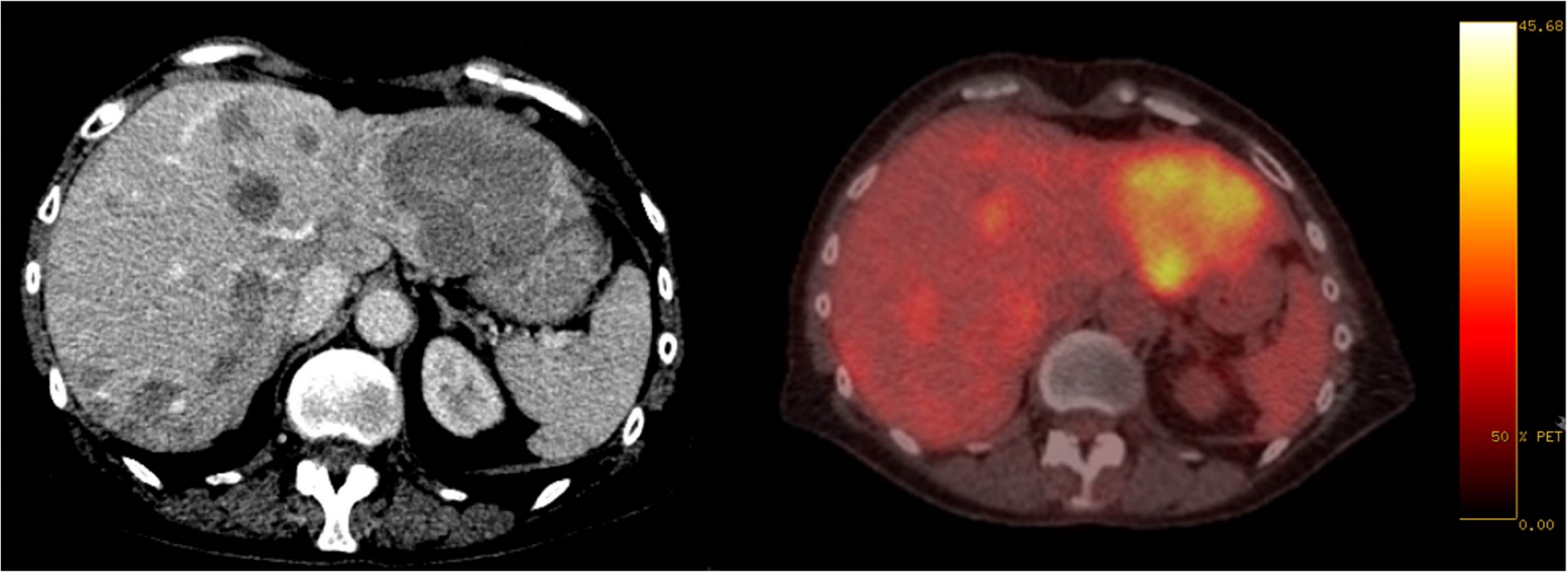
Computed tomography scan (*left*) and ^18^fluorodeoxyglucose-positron emission tomography scan (*right*) (background blood pool maximum standardized uptake value 1.5, hepatic maximum standardized uptake value 2.6) showing updated cross-sectional imaging of the patient’s liver heavily infiltrated with metastases from neuroendocrine tumor

This case was fast-tracked for discussion at the next available regional neuro-oncology and NET multidisciplinary team (MDT) meetings. Due to the location and extensive nature of the skull base metastasis, it was decided by the MDT panel that it was technically inoperable and it also could not be radically treated by stereotactic radiosurgery (SRS). Their recommendation was to consider palliative radiotherapy and further systemic management. An updated computed tomography (CT) scan was later performed confirming definite disease progression in her liver lesions compared to 3 months ago, demonstrating that CAPTEM was ineffective (Fig. 2).

The NET MDT’s consensus was to prioritize the management of the skull base metastasis, and our patient was therefore referred to the radiation oncologist. She was treated with conventional fractionated radiotherapy delivered to the base of her skull, encompassing the suprasellar region and cavernous sinus, at a dose of 20 Gy over 10 fractions. Following completion of radiotherapy, and based on the change in aggressiveness and unexpected rapid progression of the disease that she was experiencing at this point, an ^18^fluorodeoxyglucose-positron emission tomography (^18^FDG-PET) was performed.

In view of tumor positivity in the ^18^FDG-PET, intravenously administered chemotherapy with carboplatin with area under the curve (AUC) of 5 and etoposide (100 mg/m^2^) on days 1–3 per 21-day cycle was started. She completed four cycles of this chemotherapy regimen with minor radiological response. However, chemotherapy was interrupted at this point in view of clinical deterioration, which was predominantly due to Common Terminology Criteria for Adverse Events (CTCAE) grade 3 fatigue and subsequent drop in fitness (ECOG PS 3), and she was managed with best supportive care. Throughout her management, she had close contact with the hospital NET clinical nurse specialists, and had constant support from her Macmillan community nurse specialists and community general practitioners, who played a pivotal role for her and her family. She died at home 6 months after stopping chemotherapy (PFS 9 months), a year following her diagnosis of skull base metastasis.

## Discussion and conclusions

To the best of our knowledge, this is the first reported case of skull base metastasis from a well-differentiated small intestinal NET. In particular, this case also illustrates the nuanced approach of utilizing several contemporary pharmacological and non-pharmacological interventions in managing the unpredictable nature and complications of this rare disease over a relatively long time course of more than 10 years. Skull base metastases are known to be “late events” and tend to happen in patients who already have bone metastases and are more commonly seen in breast, lung, and prostate cancers [12]. Notably, our patient had skull base metastasis without known bone metastasis, but it can be postulated that her disease had disseminated hematogenously considering the large burden of her systemic disease.

Previously, there have been a few case reports which have provided further insight into the management of our case; however, these case reports described patients with NETs that originated from the skull base, some of the patients did not present with other distant metastases [13–15]. In the largest case series with 12 patients treated in a tertiary US cancer center, treatment of these primary skull base NETs was individualized, in view of the rarity and heterogeneity of the disease, and consisted of a possible combination of chemotherapy, conventional radiotherapy, and/or debulking surgery [14]. These were also reported prior to the advent of more novel targeted therapies and before the use of theranostics as standard practice in recent years, some of which had benefited our patient. As such, there is currently still no global consensus or level I evidence for the management of skull base metastases in the context of NETs. In addition, NETs rarely demonstrate significant radiological response to therapy, which makes the management of a symptomatic skull base metastasis particularly challenging. Thus, this further highlights the importance of expert discussions in an MDT meeting in this case because options had to be debated with awareness of all possible caveats. Unlike other tumor types in which administration of systemic treatment may also improve local symptoms, in our patient with NET, local therapy was therefore prioritized.

Intriguingly, skull base metastases can manifest as five different syndromes: the orbital, parasellar, middle-fossa, jugular foramen, and occipital condyle syndromes [16]. Table 1 summarizes the signs and symptoms associated with each one of these syndromes, as previously described. In retrospect, our patient had presented with parasellar syndrome whereby it is common for patients to report frontal headache and diplopia, and have ptosis, periorbital swelling, and ophthalmoplegia on examination [12, 16]. She did not have hypopituitarism, diabetes insipidus, or visual loss. This was corroborated radiologically as it was thought that the tumor was inseparable but did not invade the pituitary gland (Fig. 1). However, it is important to monitor for hormonal abnormalities which may occur following radiotherapy to the skull base.

**Table 1:** Clinical symptoms and signs of the main clinical syndromes associated with skull base metastasis

Clinical syndrome	Relative frequency	Symptoms	Signs
Orbit syndrome	12.5%	Supraorbital pain	Proptosis
		Frontal headache	Periorbital swelling
		Diplopia	Ophthalmoplegia
		Blurred vision	Decreased visual acuity
		Palpable mass	Facial numbness
Parasellar syndrome	29%	Frontal headache	Ophthalmoplegia
		Facial pain	Periorbital swelling
		Facial numbness	Visual loss
		Diplopia	Decreased facial sensation
			Hypopituitarism
			Diabetes insipidus
Gasserian ganglion syndrome	6%	Atypical facial pain	Abducens palsy
		Facial numbness	Facial palsy
		Paresthesia	
Jugular foramen syndrome	3.5%	Occipital/postauricular pain	Cranial nerve palsies (IX, X, XI)
		Hoarseness	
		Dysphagia	
Occipital condyle syndrome	16%	Occipital pain	Cranial nerve palsies (XII)
		Neck stiffness	
		Dysarthria	
Others	33%		

Extracted and adapted from [12, 16, 17]

Although rare, intracranial metastases have a negative impact on patient survival, with 40% having intracranial progression after their radiotherapy [18], and median survival after diagnosis of brain metastasis ranging between 7 to 10 months [19]. In conclusion, skull base metastases should be part of the differential diagnosis when assessing patients with long term metastatic NET with new onset of neurological symptoms. Treatment of intracranial metastasis from a rare tumor should be conducted in a multidisciplinary manner with multiple modalities if required, including surgery, radiotherapy, and systemic therapy including chemotherapy [19].

## Data Availability

All data generated or analyzed during this case study are included in this published article. Any further information can be made available from the corresponding author on reasonable request.

## Abbreviations

^18^FDG-PET: ^18^Fluorodeoxyglucose-positron emission tomography
AUC: Area under the curve
CAPTEM: Capecitabine and temozolomide chemotherapy regimen
CT: Computed tomography
CTCAE: Common Terminology Criteria for Adverse Events
ECOG PS: Eastern Cooperative Oncology Group performance status
GEP-NET: Gastroenteropancreatic neuroendocrine tumor
LAR: Long-acting release
MDT: Multidisciplinary team
MRI: Magnetic resonance imaging
NEC: Neuroendocrine carcinoma
NET: Neuroendocrine tumor
NIHSS: National Institutes of Health Stroke Scale
PFS: Progression-free survival
PRRT: Peptide receptor radionuclide therapy
SIRT: Selective internal radiation therapy
SRS: Stereotactic radiosurgery
SSA: Somatostatin analog
TACE: Transarterial chemoembolization
